# A Mobile Health App–Based Postnatal Educational Program (Home-but not Alone): Descriptive Qualitative Study

**DOI:** 10.2196/jmir.9188

**Published:** 2018-04-19

**Authors:** Shefaly Shorey, Yen Yen Yang, Cindy-Lee Dennis

**Affiliations:** ^1^ Alice Lee Centre for Nursing Studies National University of Singapore Singapore Singapore; ^2^ Department of Psychiatry University of Toronto Toronto, ON Canada

**Keywords:** parents, postnatal care, mobile applications, midwifery, nurse midwives, nursing

## Abstract

**Background:**

The postnatal period poses numerous challenges for new parents. Various educational programs are available to support new parents during this stressful period. However, the usefulness of educational programs must be evaluated to ascertain their credibility.

**Objective:**

The aim of this descriptive, qualitative study was to explore the views of parents of newborns with regard to the content and delivery of a mobile health (mHealth) app–based postnatal educational program.

**Methods:**

A qualitative semistructured interview guide was used to collect data from 17 participants who belonged to the intervention group of a randomized controlled trial. The intervention, a 4-week-long access to a mHealth app–based educational program, was evaluated. The interviews were conducted in English and at the participants’ homes. Thematic analysis was used to analyze the data. The Consolidated Criteria for Reporting Qualitative Research checklist was used to report the findings.

**Results:**

The interviews revealed 4 main themes: (1) positive features of the mHealth app, (2) advice from midwives, (3) experiences gained from using the mHealth app, and (4) recommendations for the future. The participants evaluated the educational program to be a good source of information that was tailored to the local context. The different modes of delivery, including audio and video, accentuated the accessibility of information. The parents evaluated that the facilitator of the featured communication platform, a midwife, provided trustworthy advice. Belongingness to a virtual community beyond the hospital endowed the parents the confidence that they were not alone and were supported by other parents and health care professionals.

**Conclusions:**

According to the parents, the mHealth app–based educational program was helpful in supporting a multi-ethnic sample of parents during the postnatal period. This insight indicates that the program could be implemented in a wide community of parents in the postnatal period. The helpfulness of the educational program is a testament of the potential benefits of using telemedicine among new parents postnatally. Resources can also be dedicated toward extending the duration of access to the app beyond 1 month and developing relevant content for parents across the perinatal period.

## Introduction

### Background

The postnatal period is a trying period for new parents as they adapt to the rigorous demands of parenthood [1]. During this period, parents seek to acquire new skills and find new ways of restoring balance in their lives [2]. Mothers experience challenging issues relating to health of the baby, breastfeeding, and varied sleeping patterns, which may lead to a myriad of emotions comprising self-doubt [3], anxiety disorders [4], and postpartum depression [5-8]. Although maternal morbidities have been extensively documented, fathers also experience high levels of stress while caring for the baby and adapting to their new role as a father [9]. Ineffective management of these stress levels could lead to depression [10], which may have adverse implications on child development, mother-infant interaction, as well as conjugal and family relationships [6,11]. Therefore, mitigating the development of such affective conditions in new parents through effective postnatal care and support is important.

### Current Status of Postnatal Care

Postnatal care is crucial in enhancing maternal and child well-being; however, studies have established that postnatal care from hospitals is suboptimal [12-14]. Most health services provide early discharge, that is, reduced postnatal length of stay for new mothers to reduce health care costs while sustaining the quality of care [15]. However, this practice has raised some concerns in relation to postnatal education by health care professionals, maternal confidence in transitioning to the home setting, breastfeeding, and postnatal depression [14,16,17]. New mothers also expressed great dissatisfaction with the quality of postnatal care, including insufficient time for queries to be asked, information overload within a short period of time, and an inadequate amount of midwifery care [18,19].

With current research showing the need to improve the quality of postnatal care, various interventions that aim to address parental concerns have been developed. Such interventions include domiciliary visits, clinic visits, and telephone follow-ups [20]. Home visits may be effective in providing tailored care and increasing patient satisfaction [12]. However, these measures are less feasible for health care services to adopt and implement due to logistics, such as manpower and cost [20]. Furthermore, clinic follow-up visits and telephone follow-ups usually take place on a monthly basis, which may not be solely sufficient in providing assistance to new parents during their transition to parenthood [21].

### Use of Technology in Postnatal Care

The use of technology has become a cost-effective avenue to deliver quality health care [22]. The application of information technology in enhancing an individual’s access to health care has been regarded as a promising innovation that might revolutionize health care [23]. With the advancements of technology over the decades, controlling technological innovations is important to enhance the efficiency and continuity of care during the postnatal period, thereby enhancing the satisfaction of the postnatal care provided [24]. Available literature on the use of mobile health (mHealth) apps in the postnatal period is scarce [24-27]. In addition, such literature is focused on service utilization and limited to low-middle-income countries [25-27]. The development and evaluation of mHealth app–based educational programs are encouraged from stakeholder and user perspectives [25-27].

Therefore, this qualitative study evaluated the mHealth app–based educational program catered for multi-ethnic new parents in Singapore. The focus was on exploring the parents’ perceptions of the content, strengths, and suggestions for improvements of the program.

## Methods

### Procedures and Participants

A descriptive qualitative study design was used. Semistructured interviews were conducted following the Consolidated Criteria for Reporting Qualitative Research. Participants were recruited from an intervention group of a randomized controlled trial [28]. The randomized controlled trial aimed to test the effectiveness of the mHealth app–based educational program among new parents. The participants were randomly divided into 2 groups, that is, control (receiving standardized care) and intervention (receiving educational program via the mHealth app and standardized care). English-speaking parents were invited to participate based on their parenting self-efficacy scores after the intervention. Parents with high and low parenting self-efficacy scores were purposively invited to participate in the process evaluation interviews. However, all the participants from the intervention group had parenting self-efficacy scores above the median score of 20 [28]. Therefore, all participants (50 couples, 2 fathers, and 1 mother) who completed the intervention (n=103) were approached, and 21 participants (5 couples, 7 fathers, and 4 mothers) agreed to participate subsequently. Moreover, 4 participants (3 fathers and 1 mother) subsequently refused to participate for varied reasons, such as being overseas or busy. Finally, 17 participants (5 couples, 4 fathers, and 3 mothers) were interviewed. Data saturation was achieved with 15 participants, and 2 additional interviews were conducted to ensure that no new findings emerged from the interviews.

### Intervention

Participants in the intervention group gained access to the *Home-but not Alone* mHealth app–based educational program for 4 weeks after discharge from the hospital with routine care. Routine care comprised care and support provided by nurses and midwives throughout the participants’ hospital stay, as well as a follow-up appointment with their obstetricians [12]. Various elements of the mHealth app–based educational program include a discussion forum that enabled parents to relay photographs or messages and have their queries and concerns addressed by a midwife once within 24 hours; an extensive information resource comprising audios, videos, and PDF documents on new born, maternal, and paternal care; and daily notifications received by the parents regarding their babies’ important milestones and needs. Asynchronous mode of communication was used by the midwife to answer parental inquiries once a day. The educational program follows a conceptual framework based on Bandura’s self-efficacy theory [29]. The intervention sought to be a form of social support in enhancing parenting self-efficacy and emotional well-being through reducing depressive symptoms and increasing overall parenting satisfaction. The conceptual framework is presented in the study protocol [30].

### Data Collection

After 4 weeks of access to the mHealth app–based educational program, parents were spoken to via a telephone call and were asked to participate in a qualitative semistructured face-to-face interview. The purpose was to understand their perceptions and feedback regarding the content, mode of delivery, and the effectiveness of the intervention. As both parents were given the opportunity to utilize the mHealth app, they were both eligible and invited for participation in the interview. Parents were informed that all interviews would be audiorecorded. A written, informed consent was obtained for their participation in the interviews. Finally, 5 couples, 4 fathers, and 3 mothers (n=17) participated in the interview. The interviews were conducted by the coauthor who was trained to conduct these interviews by the corresponding author. The interviews occurred at the participants’ homes, and the participants were reminded of the interview 1 day in advance via a text message. The interviews lasted between 30 and 45 min with an average time of 35 min and were carried out in English. For couples, both fathers and mothers were interviewed at the same time as most of them requested it to be that way; however, they were reminded throughout the interview to remain objective and share their personal accounts of using the mHealth app. Anecdotal notes on observational data were collected, and both fathers and mothers shared equally about their experiences in using the mHealth app. Data collection lasted from May to July 2016.

A semistructured interview guide was developed to evaluate the perceptions of the educational program delivered via the mHealth app. The guide was developed with reference to the conceptual framework of the main study [30] and was evaluated by an obstetrician, 2 midwives, and a couple from the pilot study. Probing questions were created to attain a comprehensive perspective on the effectiveness of the mHealth app. Table 1 represents the interview guide. One pilot interview was conducted to test the interview guide and the interview process, which was excluded from the final data analysis. All 17 interviews were audiorecorded and transcribed verbatim.

### Ethical Considerations

Ethical approval was acquired from the Institutional Review Board (National Health Group Domain Specific Review Board, Ref: 2015/01250) of the study hospital. Relevant information about the research study was comprehensively explained to the participants. Following which, written informed consent was obtained from each participant. Participation in this research was strictly voluntary, and confidentiality was adhered to.

### Data Analysis

Thematic analysis was performed, in which the entire dataset was reviewed and the following 3 phase analysis were used: (1) preparation of data for analysis, (2) generation of initial codes, and (3) development of respective themes and subthemes.

The coauthor interviewed the participants and transcribed the audio data verbatim without editing any grammatical errors, which is useful in retaining distinct features of the participants’ language, keeping it relevant to the local context [31]. The final transcripts were examined several times to gain familiarity with the dataset. The 2 authors were independently involved in the analysis of the content, development of the codes, and revelation of the eventual themes. An initial understanding of the content of the entire dataset was established.

Subsequently, the color-coding method was used to identify different concepts, forming the initial codes. These excerpts were then extracted and placed into a tabular format, entailing themes and subthemes within a new document. The authors reviewed the themes for likeness and differences before deciding the overarching themes. To achieve confirmability and objectivity in the analysis of data, the various themes and subthemes were discussed extensively through several meetings between the 2 authors. Any discrepancy was discussed with the third author. The field notes were often referred to, and constant comparative analyses were performed. Member checking was also established with the participants after each interview by allowing them access to their transcripts to ensure that the intended meanings behind their verbatim quotes were accurately captured. The final findings were shared with the participants, and they had no further inputs on the derived themes and subthemes.

### Rigor

To enhance credibility and trustworthiness of the study, reflexivity was maintained throughout the data collection and analysis [32]. Field notes were taken to capture nuances in nonverbal responses from the participants and to enhance the authenticity of the verbal quotes. The coauthor maintained a diary and constantly self-reflected before and after the interview to maintain accuracy in reporting the participants’ emic views during the data collection. The purpose of these reflections was to consciously acknowledge one’s values, assumptions, and goals toward the study topic so that the researchers can clarify their belief systems and subjectivities toward participants’ responses. A semistructured interview guide was developed based on a theoretical framework and was evaluated by the experts and the parents.

**Table 1:** Process evaluation semistructured guide. mHealth: mobile health.

Number	Probing question
1	What were some of the education or services that you received in the hospital to better prepare you for participation in your baby’s care?
2	What other types of services have you participated in or received apart from those provided in the hospital?
3	Did you find the *Home-but not Alone* mHealth app useful? Probe: If yes, how? If no, why not?
4	What are the strengths of the *Home-but not Alone* mHealth app? Probe: Do you think the duration of the access to the mHealth app was sufficient?
5	What were the main difficulties that you faced while using the *Home-but not Alone* mHealth app? Probe: Can you describe any technical difficulties you might have faced? What would you want to improve about the app?
6	Do you think it was appropriate to introduce the mHealth app after childbirth? Probe: Would you want to have the app made available for your future babies? Do you think such an app should be made available during pregnancy too?
7	Do you think use of the *Home-but not Alone* app has enhanced your parenting confidence? Probe: If yes, how? If no, why not?
8	Do you think use of the *Home-but not Alone* app has enhanced your parenting satisfaction? Probe: If yes, how? If no, why not?
9	Were you satisfied with the added support you received in terms of the mHealth app? Probe: If yes, how? If no, why not?
10	Did you feel emotionally supported and stable with the additional support you received from the mHealth app? Probe: If yes, how? If no, why not?
11	Would you recommend the use of the *Home-but not Alone* mHealth app to others?

## Results

### Sample Characteristics

A total of 5 couples, 4 fathers, and 3 mothers were interviewed. Their ages ranged between 26 and 42 years. Among them, 41% (7/17) were Chinese, 29% (5/15) were Malay, 6% (1/17) were Indian, and 24% (4/17) were of other ethnicities. The majority were degree holders and full-time employed staff at various organizations. The majority (13/17, 86%) of participants had a monthly household income of more than SG $6000. Nearly half of the participants attended antenatal classes before delivery, and 71% (12/17) experienced a normal vaginal delivery. All the participants were married. A description of the participants’ characteristics is displayed in Multimedia Appendix 1.

Four major themes emerged from the thematic analysis of the interviews: (1) positive features of the mHealth app, (2) advice from the midwife, (3) experiences gained from using the mHealth app, and (4) recommendations for the future. The themes and subthemes are summarized in Textbox 1.

### Theme 1: Positive Features of the mHealth App

Most of the parents found that the mHealth app was a good informational resource that catered to the local context and new-generation parents, and that the information provided was tailored to individualized needs, easy to access, and allowed the recap of information.

#### Subtheme 1: Good Informational Resource

Many parents utilized the mHealth app as a point of reference to clarify any doubts they faced when performing routine baby care tasks. The participants compared the mHealth app with a library, regarding it as a useful informational tool in preparing them for any issues that they might potentially experience during the first few weeks:

It’s like a library for me…I can just view it to see what new parents face at this point of time.

Participant 3

#### Subtheme 2: Cater to Local Context and New Generation Parents

Many parents discerned the mHealth app to contain localized information. Thus, the app is more contextually relevant as opposed to other global apps. These data include the matrices used (cm and kg vs feet and pounds) and the discussion of common issues babies encounter (Asian vs Caucasian):

I think the app is better, because, I say, even the matrix uses cm and kg...I would say the app is more appropriate and suitable for Asian mummies, especially in Singapore.

Participant 8

#### Subtheme 3: Tailored to Individualized Needs

The parents felt that being able to acquire answers from the midwife and having their queries answered through the discussion forum provided a clearer and more individualized answer compared with the standard answers found on web:

Yes, [any answer found] on web is vague and very generic [in their] answer. For example, each baby should be gaining this certain amount of weight when you are pregnant, so you like, okay, [but] when you go to the doctor, [you realize,] oh no, your baby is not gaining as much as what [the] web recourses have been saying…but with this app, we received focused answers to our queries.

Participant 11

#### Subtheme 4: Various Features—Audio, Video, Push Notifications, and Discussion Forum

The mHealth app catered to the participants’ individual preferences of learning styles through the different modes of educational materials. Some mothers found the audio files more convenient while breastfeeding. Others perceived the videos to be an easier way to learn hands-on skills. A good mix of both theory (PDF files and audios) and practical (videos and the discussion forum) learning existed, which the parents could benefit from:

...but you know when you breastfeed, sometimes you feel tired, you want to [be] lying down, but breastfeeding and lying down is sometimes [a] problem. So, I realized that there are audios for each of the PDF file. So, I just stuck my earphone [in], and listened to it.

Participant 8

#### Subtheme 5: Easy to Access and Allows Recap and Recall

Majority of the parents expressed that the interface of the mHealth app was user-friendly, making it easy to acquire necessary information through their phones. Many participants also revealed that the mHealth app facilitated their recapping and recalling of essential information required in taking care of their baby:

...because, when you learn at the hospital, you only see [it] one time. If you watch the video, you can recap.

Participant 7

### Theme 2: Advice From the Midwife

The participants underlined the advice given by the midwife to be reliable, reassuring, prompt, and that it also facilitated their decision-making process.

**Themes and subthemes of the findings.**

**Positive features of the mobile health (mHealth) app**

1. Good informational resource
2. Cater to local context and new generation parents
3. Tailored to individualized needs
4. Varied features: audio, video, push notifications, and the discussion forum
5. Easy to access and allows recap and recall

**Advice from the midwife**

1. Reliable guidance
2. Reassuring advice
3. Prompt replies
4. Facilitation of the decision-making process

**Experiences gained from using the mHealth app**

1. Provides continuity of care
2. Is like a friend
3. Integrates a community of people who are undergoing a similar phase in their lives, that is, parenthood
4. Enhances confidence
5. Enhances satisfaction
6. Receptiveness

**Recommendations for future**

1. Reduce technical hiccups and enhance technical aspects
2. Extend the duration of use of the mHealth app
3. Encourage engagement with the mHealth app
4. Widen educational topics

#### Subtheme 1: Reliable Guidance

Nearly all the participants felt that they can easily trust the advice provided by the midwife as opposed to acquiring information on web where sources may not be as credible:

...being able to get the response you knew you could trust such as a midwife…Er…giving us advices, you could trust that.

Participant 10

#### Subtheme 2: Reassuring Advice

The advice given by the midwife often left parents reassured and empowered to overcome the issues they encountered. The participants could even resonate with the advice given for queries other than that of their own:

I think the best part about it is the personal touch…from the midwife herself. When you ask questions, and know that the midwife will always reply with a ‘you’re doing good mummy keep it up!’ [laughs] Yea, so I think it is encouraging…even though it is not me who sent the question. But when you read [it]…you know? That’s nice.

Participant 17

#### Subtheme 3: Prompt Replies

The participants were also content with the efficiency of the midwife’s replies to their queries, which were useful in alleviating their worries within a relatively short period of time:

The questions we were asking also, they were answered very promptly. I think that was quite amazing. I think it was…not even within 24 hours, but I think within 12 hours…we will get a reply also…

Participant 12

#### Subtheme 4: Facilitation of the Decision-Making Process

Several parents felt that the midwife provided detailed explanations, thereby giving them improved understanding of their situation. This initiative allowed them to make informed decisions regarding the care of their baby:

...after seeking midwife’s advice, that’s when I decided to bring the baby for his jaundice check…because of the answers that have been given to us, lah. So, it reassured me to seek help.

Participant 3

### Theme 3: Experiences Gained From Using the mHealth App

The experiences gained by most participants entailed the continuity of care received, the integration of a community of people undergoing a similar phase in their lives, and the increased confidence and satisfaction of taking care of their babies.

#### Subtheme 1: Provides Continuity of Care

The continuous support received by the parents via the mHealth app was gratifying because it facilitated easy transition from the hospital setting to their home. Many participants also felt comforted and secure in having their queries answered by other experienced parents:

I think what is good about the app is [that] you have the mothers and fathers for users, user-based answering the questions, but then in 9 out of the 10, the moderator [midwife] comes on and either clarifies all the answers other people [have] given, and adds an extra professional part as well.

Participant 9

Several parents felt that the app acted as a support mechanism between the doctor’s follow-up sessions, which typically lasted between a week to a month, thus providing comfort and reassurance to the parents regarding their concerns:

I really feel that…not to worry…too much. Because you can only meet the doctor at [a] certain point in time, right? So, in the meantime, we didn’t have any information. So we were glad that we had the message.

Participant 5

#### Subtheme 2: Is Like a Friend

The mHealth app was described as a friend to new parents by providing much-needed support during their transition phase, reassuring them that they were not alone:

I say, “friend”…Because sometimes you were alone, then…I went to the mobile app a lot to see what other mums said.

Participant 14

#### Subtheme 3: Integrates a Community of People Undergoing a Similar Phase in Their Lives, That Is, Parenthood

The majority of the parents emphasized that the mHealth app helped them to realize that the issues they were facing were commonly experienced by many parents, thereby putting their problems into perspective and allowing them to feel better. Most participants appraised the expediency of learning from the experiences of other parents that were shared within the discussion forum or by using their own experiences to help answer other parents’ queries:

So when we have this app and you know there’s another parent…the person answering is really answering related directly to the question. And the question is linkable to us…We felt others are also going through the same things…So I think that was the most helpful about the app.

Participant 17

The participants exhibited mutual empathy, which is known to augment prosocial behaviors, such as supporting one another. This value was commonly manifested in the form of multiparous parents being willing to share their past experiences to alleviate the worries and concerns of their primiparous counterparts:

I have been through this…I know how it feels to be a new mother…So, if I [can] confirm [that] I know the answer, then I will try to help out by answering the queries.

Participant 13

#### Subtheme 4: Enhances Confidence

The educational materials and having their queries answered by the midwife reassured many participants that they were doing the right thing for their baby:

Because when I asked the question and then I’ve got [a] response [that] I was comfortable knowing, doing the right thing. I can continue that way, and you feel more confident when you are making [the] right decisions.

Participant 10

#### Subtheme 5: Enhances Satisfaction

Many parents expressed a sense of satisfaction in relation to their parenting role with the support from the mHealth app and felt they were more adept in playing their parenting role:

Er…I think, with the increased confidence level, then you’ll be more satisfied…Because you feel you understand your child better! And you know when this happens…you know roughly what to do.

Participant 17

#### Subtheme 6: Receptiveness

All the participants were receptive to using the mHealth app in the future if they have another child and were forthcoming to recommend this mHealth app to their friends who were expecting:

The Home-but not Alone, I am going to recommend because [it is] very focused on [the] first month of the support. We need it.

Participant 5

### Theme 4: Recommendations for Future

Many parents disclosed that certain technical hiccups should be resolved and enhancements should be added in the mHealth app. In addition, the duration of use of the mHealth app needs to be extended, the usability of the mHealth app has to be promoted, and additional educational topics included.

#### Subtheme 1: Reduce Technical Hiccups and Enhance Technical Aspects

Some participants felt that the language in the video was not clearly audible due to the enunciation and suggested for the addition of subtitles. The majority of the parents highlighted the need to add notifications to their queries because checking repeatedly for any replies to their raised queries was time-consuming. Furthermore, many participants found locating specific information within the discussion forum very tedious because questions were not organized into topics:

Yea, because now I only look based on the latest post, but not what is actually relevant…I will have to open each one [discussion thread] to read and see if there’s anybody talking about breastfeeding.

Participant 14

#### Subtheme 2: Extend the Duration of use of the mHealth App

Participants felt the need to extend the duration of the mHealth app by introducing the mHealth app during the antenatal period, preferably during the third trimester to prepare themselves:

I think during the third trimester…Yea…towards the third trimester, I think you’re more ready and prepared to actually absorb all the knowledge.

Participant 4

Most participants also conveyed their interest in using the mHealth app for more than a month after the baby was born and requested for information to be provided for important milestones within the first 6 months of their baby’s growth, such as at 3 and 6 months:

I think maybe the first six months…Because it’s the first vital…you know…stages! After that, I think they can start crawling, recognizing you proper[ly], so it shouldn’t be so much of a problem.

Participant 17

#### Subtheme 3: Encourage Engagement With the mHealth App

Participants felt the need to promote members of the group to be active users of the mHealth app and to ask questions. They believed that the number of questions asked translated into how much they can learn from:

...but I think it would have been even more useful if more people shared…because different people had their [own] way of coping.

Participant 12

#### Subtheme 4: Widen Educational Topics

Fathers faced concerns regarding their roles in supporting their wives emotionally during this time and requested for such relevant topics. Many parents also felt that the current topics found in the mHealth app mostly catered to first-time parents. They felt that the mHealth app would be more beneficial if it included topics relevant to multiparous parents, such as dealing with older siblings and babies:

...because some of the other topics I have already read about or did research on my first baby. But, now that it is my second baby, it might be useful to have some topics for parents that are already parents.

Participant 16

## Discussion

### Principal Findings

The participants reported numerous strengths of the app. Many parents expressed that it was convenient and found that it encapsulates a wide range of educational information, which allowed for an easy search and retrieval of information independently. Such information was useful as a general informative resource or for more explicit information that may address their specific concerns. A previous research [33] found that mothers preferred midwives to provide information in a nondirective form to allow them the opportunity of making informed decisions independently. Similarly, the app facilitated parents in their decision making to a certain extent and encouraged them to act autonomously. This function is in accordance with Bandura’s theory of self-efficacy [29], in which learning independently and building one’s own experiences is one way to attain the mastery of such experiences, thereby augmenting parenting self-efficacy [24].

The multifaceted approach regarding the delivery of information through PDF, video, or audio files augmented the expediency of the app and enhanced parenting self-efficacy. Mothers were able to breastfeed and listen to audio recordings, and the parents reported that they were only able to observe demonstrations on baby care tasks once within the hospital; hence, the videos came in as a handy tool for recapping and recalling practical tasks as opposed to written information. A previous study acknowledged that the memory for visual depictions is greater than that for verbal or written content [24]. Moreover, the learning theory [34] asserts that learning is more enhanced when the content is organized as opposed to being fragmented. Moreover, according to Bandura [29], vicarious encounters can instill in parents a belief that they too have the ability to carry out the task at hand, which can lead to great parenting self-efficacy.

The participants also expressed their satisfaction toward the app in that it provided a means of access to support from a health care professional. Receiving guidance from a health care professional is a vital facet in parents developing a sense of security during the postnatal period [24,35]. The answers to their queries by the midwife were regarded as trustworthy, prompt, and reassuring. This finding corroborates with a previous study that regarded midwives as a point of reference when making decisions [2]. It implies the usefulness of asynchronous communication in alleviating concerns for new parents in their transition into parenthood.

The app was also beneficial in creating a community in which parents could learn from the issues and concerns brought up by other parents as well as receive advice from those with experience. As previously noted [33], mothers found sharing their experiences with one another and gaining reassurance and support beneficial. Although sharing with a professional would generate a clinical or scientific reasoning on what was being physically experienced by them, sharing with other mothers produced a communal response of the emotional experience itself. Although advice from other experienced parents was easily available, it was constantly reviewed by the midwife and, in case of any discrepancy, the advice was corrected. In general, experienced parents’ advice was appropriate and practical, and overall, parents also appreciated a conducive milieu to express their concerns and felt validated.

The app was successful because most participants no longer felt alone but emotionally supported by both the midwife and other parents. Sharing their experiences with other parents who are undergoing similar encounters can create a sense of belonging within a particular community as well as enhance their social support. Both factors are essential in enhancing parents’ psychological well-being [23]. Similarly, previous studies [36,37] demonstrated that the midwife offers emotional support to parents as opposed to providing support that is solely informative or didactic, which is commonly observed among health care professionals. The finding that parents can feel supported through the mHealth app implies that personal encounters or direct communication is no longer the only way for individuals to gain emotional support; moreover, with the rise in the utilization of technology and social media, information technology might be an alternative means of extending emotional support [23].

All participants demonstrated their keenness to use the app in future childbearing or to recommend it to their friends who might be expecting, showing their receptiveness and satisfaction toward using this app. This receptivity may be attributed to the essence of anonymity that comes with using information technology, enabling parents to ask questions openly without feeling embarrassed about their uncertainty [24].

The participants felt more confident in performing parenting tasks because they felt adequately guided through the videos and the support they received from the midwife and other parents. Most participants also expressed that the midwife was very encouraging and reassuring in her feedback, which instilled in them the confidence to perform the tasks at hand. According to Bandura’s theory of self-efficacy [29], verbal persuasion is another way to enhance self-efficacy. Individuals who are continually reminded that they harness the ability to become proficient at various tasks will be more likely to muster up the courage and effort to work toward achieving it [34,38].

### Strengths and Limitations

This study is the first to evaluate the technology-based postnatal educational program qualitatively among the multiethnic parents in Singapore. The qualitative approach provided the in-depth perspectives of new parents in receiving the mHealth app–based educational program. This study also has few limitations. It may not be representative of the entire population because a high proportion of the participants were of high economic status (employed, educated, and had high income). In addition, the study focused only on English-speaking parents. Future studies should include multilingual participants with differing socio-economic backgrounds. Moreover, only parents’ perspectives were captured in the usefulness of the program. Future research could explore the views of the involved midwives or health care professionals regarding the feasibility or acceptability of such a program in their professional practice.

### Conclusions

Technological advancement and social media usage provide the impetus for health care to invest in the development of information technology to render accessible and efficient care. This research provides insights into the use of technology in providing support to new parents during the postnatal period. Specifically, it details parents’ perceptions on the content and effectiveness of the mHealth app on their satisfaction and well-being. Future studies should continue to evaluate the use of information technology, especially across the perinatal period.

## Appendix

Multimedia Appendix 1 Demographic details of participants who participated in process evaluation interviews.

## Abbreviations

mHealth: Mobile Health
RCT: randomized controlled trial
