# Supplementary material for: A Mobile Health App–Based Postnatal Educational Program (Home-but not Alone): Descriptive Qualitative Study
Source: J Med Internet Res. 2018 Apr 19;20(4):e119. doi: 10.2196/jmir.9188 (PMC5934535; doi:10.2196/jmir.9188)
Supplement: Multimedia Appendix 1 [file jmir_v20i4e119_app1.pdf]

Appendix

Table A: Description of participants for process evaluation (n=17).

**Participant**	**Age**	**Marital Status (M[a], M-C[b])**	**Gender**	**Ethnicity**	**Highest educational level**	**Employment**	**Monthly household income[d]**	**Antenatal class attendance**	**Types of birth (NVD[e], assisted, LSCS[f])**
1	31	M-C	Male	Chinese	ITE[c]	Yes	SG[g] $3000–SG $5999	No	NVD
2	26	M-C	Female	Malay	Diploma	Yes	SG $3000–SG $5999	No	NVD
3	26	M-C	Male	Malay	Diploma	Yes	SG $3000–SG $5999	No	NVD
4	34	M-C	Female	Malay	Degree	Yes	SG $3000–SG $5999	No	NVD
5	38	M-C	Male	Chinese	Degree	Yes	SG $6000–SG $9999	Yes	LSCS
6	33	M-C	Female	Chinese	Degree	Yes	SG $6000–SG $9999	Yes	NVD
7	29	M-C	Male	Malay	Degree	Yes	SG $6000–SG $9999	No	NVD
8	28	M-C	Female	Malay	Degree	Yes	SG $6000–SG $9999	No	NVD
9	35	M-C	Male	Chinese	Degree	Yes	>SG $10,000	Yes	NVD
10	33	M-C	Female	Chinese	Degree	Yes	>SG $10,000	Yes	NVD
11	35	M	Male	Others	Degree	Yes	SG$6000–SG$9999	No	NVD
12	33	M	Female	Indian	Degree	Yes	>SG$10,000	No	NVD
13	42	M	Male	Others	ITE	Yes	>SG$10,000	Yes	LSCS
14	30	M	Female	Others	Degree	Yes	SG$6000–SG$9999	Yes	LSCS
15	30	M	Male	Chinese	Degree	Yes	SG$6000–SG$9999	Yes	Assisted
16	26	M	Female	Chinese	Degree	No	SG$6000–SG$9999	Yes	Assisted
17	40	M	Male	Others	Degree	Yes	>SG$10,000	No	NVD

[a]M=Married.

[b]M-C=Married and participated as a couple in this study.

[c]ITE=Institute of Technical Education.

[d]Calculated in Singapore Dollar.

[e]NVD=Normal vaginal delivery.

[f]LSCS=Lower segment caesarean section.

[g] SG = Singapore.
